# Confining a Protein-Containing Water Nanodroplet inside Silica Nanochannels

**DOI:** 10.3390/ijms20122965

**Published:** 2019-06-18

**Authors:** Lara Giussani, Gloria Tabacchi, Salvatore Coluccia, Ettore Fois

**Affiliations:** 1 Dipartimento di Scienza e Alta Tecnologia and INSTM udr Como, Insubria University, Via Valleggio 9, I-22100 Como, Italy; largius13@icloud.com (L.G.); gloria.tabacchi@uninsubria.it (G.T.); 2 Dipartimento di Chimica, Turin University, Via P. Giuria 7, I-10125 Turin, Italy; Salvatore.coluccia@unito.it

**Keywords:** host–guest systems, protein confinement, molecular dynamics, mesoporous silica, water nanodroplets

## Abstract

Incorporation of biological systems in water nanodroplets has recently emerged as a new frontier to investigate structural changes of biomolecules, with perspective applications in ultra-fast drug delivery. We report on the molecular dynamics of the digestive protein Pepsin subjected to a double confinement. The double confinement stemmed from embedding the protein inside a water nanodroplet, which in turn was caged in a nanochannel mimicking the mesoporous silica SBA-15. The nano-bio-droplet, whose size fits with the pore diameter, behaved differently depending on the protonation state of the pore surface silanols. Neutral channel sections allowed for the droplet to flow, while deprotonated sections acted as anchoring piers for the droplet. Inside the droplet, the protein, not directly bonded to the surface, showed a behavior similar to that reported for bulk water solutions, indicating that double confinement should not alter its catalytic activity. Our results suggest that nanobiodroplets, recently fabricated in volatile environments, can be encapsulated and stored in mesoporous silicas.

## 1. Introduction

The interaction of biologically relevant molecules with inorganic surfaces is a key ingredient in a broad diversity of scientific and technological fields, encompassing, for example, prebiotic chemistry and its intriguing relationships with the origins of life [1,2,3], biomedical applications [4,5,6,7] such as the realization of biomaterials [8] and drug carriers targeting specific organs [4,5,9,10], or the possibility of utilizing enzymatic catalysis under conditions different from those typical of physiological environments [11,12,13].

The ubiquitous, cheap, and environmentally friendly silica is a prominent material to investigate and exploit the interactions of proteins with surfaces [2,14,15]. Hydrophilicity in silica surfaces may be tuned by varying surface concentration of silanol groups (Si–OH)^−^ which impart hydrophilic functionality, and siloxane bridges (–Si–O–Si–), which are normally considered hydrophobic [16,17]. Nonetheless, in aqueous solutions characterized by pH values greater than 3, silica surfaces are negatively charged, because the silanol groups can be present in their deprotonated silanolate form (Si–O^−^) [16]. 

The incorporation of bioorganic systems inside a porous silica matrix [18] has been long recognized as a route to organic–inorganic hybrid materials endowed with new functionalities. In this way, the confining environment of the pores could not only protect the guest molecules in hostile environments, but also favour the formation of organized supramolecular systems with technologically appealing collective properties [19]. This kind of strategy has been successfully applied to the encapsulation of molecules in crystalline microporous hosts, producing hybrid devices now actually exploited in solar energy harvesting [20,21,22,23,24] and theranostics [25,26,27,28]. On the other hand, the encapsulation of a macromolecular system, such as a protein, requires larger pores size, hence the need of mesoporous silica matrices [14,29]. To this aim, it is particularly convenient to use mesoporous silicas like MCM-41 [30] and SBA-15 [31], which have hexagonal arrays of non-intersecting one dimensional channels and tunable nanosized pore openings. These hosts also enable to study the fundamental features of the interactions between proteins and silica surfaces [12,32,33,34], which are an issue of increasing relevance for both basic and applied science. As is well known, water plays a key role in mediating molecular interactions at silica surfaces [19]. Experimental and theoretical studies of bio-inorganic hybrids are normally performed in aqueous systems because water helps preserving the structure and biologic functionality of proteins [16,35,36,37,38]. Actually, the confinement of biosystems within nanosized water aggregates is by itself of high current interest, as it has recently emerged as a new technology to experimentally detect conformational changes of protein active sites [39].

In this rapidly advancing field, modeling is crucial for understanding at molecular-level the interactions of biomolecules with silica [2,40] in the presence of water. Indeed, theoretical studies on proteins at silica interfaces have experienced an impressive growth in the last few years [2,41,42,43,44], uncovering important microscopic features of the interaction with flat surfaces. The simulation of lysozyme [45,46], papain [47], and fragments of various proteins [48,49,50] on silica surfaces, for instance, revealed conformational changes, and in some cases even a certain degree of unfolding of the biomolecule upon surface adhesion. Because of the large size of the models, these calculations are normally performed with molecular dynamics and classic force field approaches [40]. In a different way, a more accurate description may be obtained by considering amino acid molecules on silica surfaces, and, more recently, in MCM-41 pores, for which quantum chemical methods can be employed [2,51]. Several quantum chemistry and experimental investigations have highlighted significant interactions of amino acids, drugs, and other organic species in contact with SiO_2_-surfaces [41,52,53,54,55,56]. Also, first principles molecular dynamics of nanoconfined organic molecules has enabled a deeper understanding of the key influence of water on the stability of hybrid functional materials [57,58,59,60]. 

In spite of these advances, the theoretical study of biopolymers/mesoporous silica composites remains a fascinating challenge because of the inherent complexity of these materials. One of the problems is that the size of the protein is typically very close to the internal diameter of the mesopores: hence, tight confinement might obstacle the reorientational dynamics of the biopolymer with respect to solution conditions [61]. However, porcine pepsin [62], a globular enzyme consisting of 326 residues (*M* = 34,623 kDa) of 4.5 × 5.0 × 6.6 nm^3^ size under encapsulation conditions [63,64], has been successfully incorporated in the circa 7.0 nm diameter channels of SBA-15, providing evidence that the encapsulated porcine pepsin maintained its catalytic functionality [31]. 

Importantly, the study of confined water and of water nanostructures has gained increasing momentum in the recent years, from both experimental and modelling investigations [65,66,67,68,69,70,71,72,73]. It is well established that these exotic phases of water exhibit unusual physicochemical and structural properties [74,75,76,77,78,79,80,81,82]. Water nanodroplets, for example, can maintain the liquid state at temperatures well below water freezing point at normal conditions [65], and exhibit intriguing behaviors—from evaporation to droplet lift-off—when in contact with oscillating surfaces [66]. These properties might also be exploited to gather a deeper knowledge of biomolecules in nanoscale confinement.

Indeed, very recently, the confinement of proteins in water nanodroplets has emerged as a unique way to investigate protein dynamics. Specifically, laser irradiation of ubiquitin protein embedded in a water nanodroplet, formed in an electrospray environment, followed by mass spectroscopy analyses revealed a sort of melting-like phase transition of the protein within the droplet [39]. This approach provided a detailed picture of the first stages of the protein denaturation process [39,83], and, in a broader perspective, represents an exciting opportunity to investigate structural changes of biomolecules taking place in confining environments. Also very recently, the simulation of water nanodroplets on surfaces exhibiting hydrophilic/hydrophobic patches revealed unidirectional water transport at ultrafast velocities (10^2^ m/s), opening new potential avenues in directed nanoscale drug delivery [84].

Nonetheless, the fundamental challenges of such a novel and rapidly-evolving field were anticipated, some years ago, by the exploration, via molecular dynamics simulations, of the behavior of a protein-containing water nanodroplet on a flat silica surface [85]. There, we highlighted the effects of the surface stoichiometry on the adhesion capability of a pepsin-containing water nanodroplet. Now we focus on a similar water nanodroplet, which incorporates pepsin and is, in turn, encapsulated inside a cylindrical silica pore mimicking the interior of a channel typical of SBA-15, thus providing a protective environment to the nanobiodroplet. We chose pepsin because experimental results evidenced that this specific enzyme can be successfully enclosed inside the SBA-15 nanochannels without loss of catalytic efficiency. This supports our idea to model the behavior of a pepsin-containing water nanodroplet of diameter compatible with SBA-15, which might be confined and stored in this mesoporous material for further studies aimed at the investigation of its behavior. 

In this work, our specific aim is to model this caged nano-bio-droplet and clarify the nature of the forces relevant for either adhesion or diffusion within a cylindrical pore with either hydrophobic or hydrophilic character. The effect of the nanodroplet size on the adhesion behavior will be also considered, by examining whether confinement in a smaller droplet (i.e., partial dehydration) could lead to conformational changes of the protein. We will show that the nanobiodroplet adheres only to negatively charged regions of the pore surface. The adhesion occurs with no direct protein–silica contact, because the interaction is mediated by water molecules and counter ions. The indirect, weak protein–surface contact, maintained also in the partially dehydrated system, is instrumental to keep the structure of the catalytic site unaltered and to conserve the enzyme functionality within the water nanodroplet.

## 2. Results and Discussion

**Figure 1:**
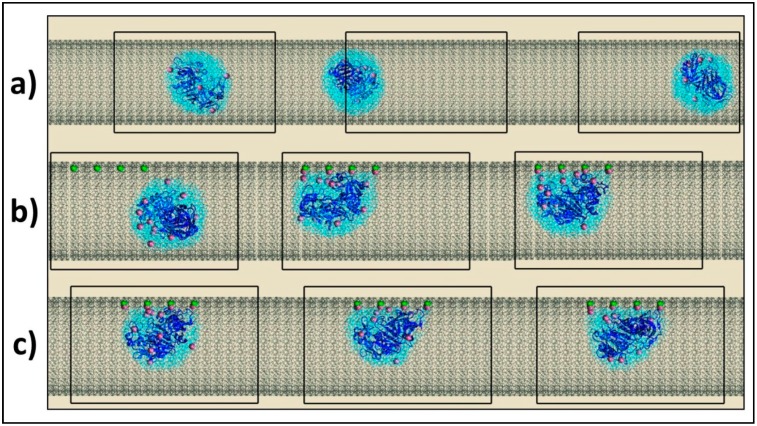
(**a**) Snapshots for evenly sampled configurations (time is growing left to right) from the simulation with a neutral cylindrical mesopore (Model 1). (**b**) Snapshots taken from the simulation with a charged mesopore (Model 2). (**c**) Snapshots taken from the simulation with a negatively charged silica channel and a lower number of water molecules (Model 3). The mesoporous matrix is shown as gray lines. Deprotonated silanols and potassium cations are represented as green and purple spheres respectively. Water molecules are represented as light blue lines. Pepsin is represented as a blue ribbon structure. The simulation box (in the yz plane) is shown as black lines.

Let us consider first the behavior of the protein containing water nanodroplet inside a neutral (hydrophobic) mesopore (Model 1). The droplet is not firmly adsorbed on the matrix: it flows through the tube weakly interacting with the hydroxylated surface, as shown in Figure 1a. On the other hand, when the nanobiodroplet is hosted in a negatively charged mesopore (Model 2), a different behavior is observed, as evidenced in Figure 1b. Like in the case of flat negatively charged silica surfaces [85], the charge compensating potassium cations bind to the deprotonated silanol groups and drag the pepsin containing droplet toward the charged region of the pore. As an effect, the droplet shape changes significantly, because water spreads over the charged patch on the mesopore surface. However, as already observed with the silica slab model, the biopolymer remains inside the water droplet and is still fully solvated.

**Figure 2:**
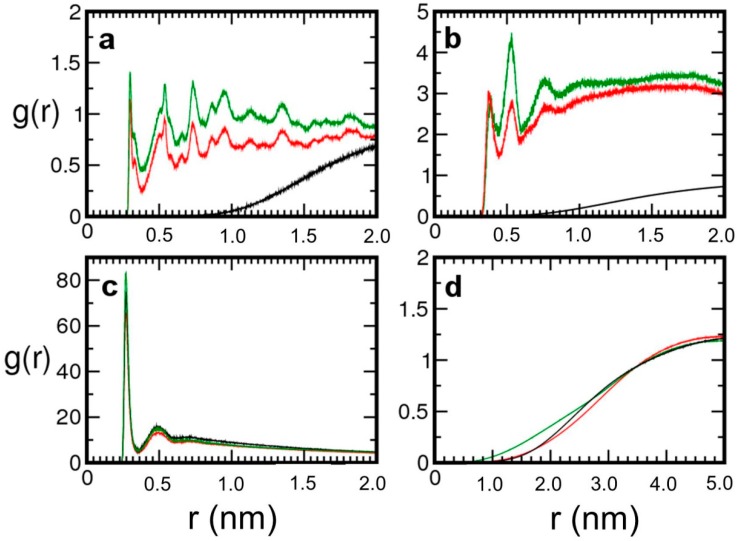
Radial distribution functions g(r) calculated for the simulations with the neutral channel (Model 1, black lines), with the charged channel (Model 2, red lines) and with the charged channel and a lower number of water molecules (Model 3, green lines). Panel (**a**) surface silanols–potassium ions radial distribution functions; panel (**b**) Silanols-water oxygen (Model 1) and silanolates–water oxygens (Models 2 and 3) radial distribution functions; panel (**c**) water oxygens–cations radial distribution functions; panel (**d**) surface silanols–pepsin atoms radial distribution functions.

To further investigate the water-mediated protein surface interactions, a model (Model 3) consisting of a charged mesopore and a smaller water droplet was simulated (see Figure 1c). A behavior similar to Model 2 was observed: the protein was still solvated and the water bio-droplet adhered to the charged region of the pore inner surface, drawn by the potassium cations adsorbed on the deprotonated silanols. Not surprisingly, by decreasing the nanodroplet size, the average protein–surface distance got smaller, as indicated by the corresponding pair distribution functions (g(r)’s), depicted in Figure 2.

**Figure 3:**
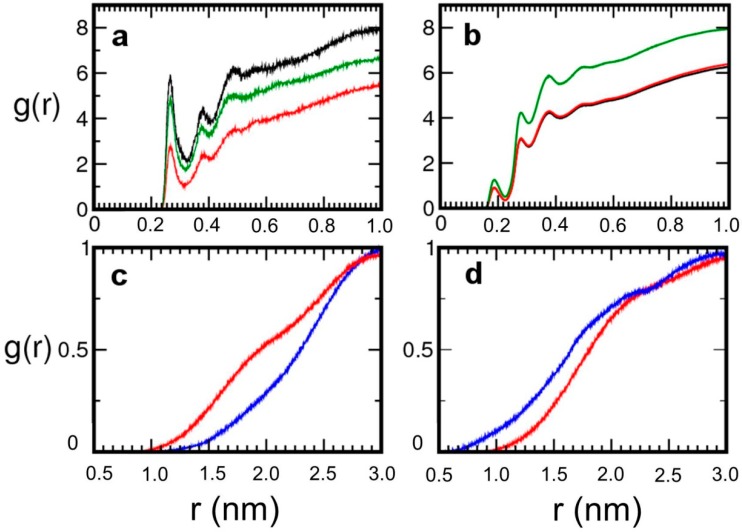
Radial distribution functions g(r) calculated for the simulations with the neutral channel (Model 1, black lines), with the charged channel (Model 2, red lines) and with a lower number of water molecules (Model 3, green lines). Panel (**a**): pepsin atoms—cations radial distribution functions; panel (**b**): pepsin–water oxygen atoms radial distribution functions; panel (**c**): radial distribution functions between the charged protein residues and surface oxygens from Model 2; panel (**d**) and Model 3. The g(r)’s for positively and negatively charged residues are in blue and red, respectively.

Binding of potassium cations to the negatively charged surface sites is confirmed by the K^+^- silanol oxygens pair distribution functions obtained for Model 2 and Model 3 (Figure 2a), both characterized by a first maximum at 0.298 nm, i.e., very close to the position found for the negatively charged silica slab (0.300 nm). Also, the water-silanolate and water potassium g(r)’s suggest that the protein–inorganic matrix adhesion should be driven by the same mechanism observed with the flat surface: namely, K^+^ bound to silanolates attract water (Figure 2b) which, in turn, draws the enzyme towards the negatively charged regions of the pore surface. The K^+^-O_WATER_ pair distribution functions (Figure 2c) indicate that potassium ions are fully solvated by the water droplet and interact with pepsin (Figure 3a).

On the whole, pair distribution functions analysis shows that the electrostatic attraction between cations and the charged surface regions (red and green lines in Figure 2a), mediates the interaction between the pore and the nanodroplet, which still solvates pepsin (Figure 3b) allowing thus for a labile adhesion of the protein to the pore inner surface. The radial distribution functions between pepsin and silica surface atoms in Figure 2d confirm that pepsin gets closer to charged surfaces, in particular in the case of the partially dehydrated system (Model 3), and also demonstrate that, at these conditions, there is still no direct contact between protein and inorganic matrix. Indeed, the average minimal inter-atomic distances between the enzyme and the inorganic support, calculated from the performed simulations, are 0.647 ± 0.208 nm, 0.525 ± 0.152 nm, and 0.326 ± 0.051 nm for Model 1, Model 2, and Model 3 respectively.

The significantly lower standard deviation value obtained for Model 3 suggests that the protein motion becomes more hindered in a smaller nanodroplet. Protein charged side chain groups are not in direct contact with surface silanols as indicated by the g(r)’s in Figure 3c,d. However, whereas in Model 2 the negatively charged residues are closer than the positively charged residues to the silanol groups, the opposite behavior is observed in Model 3, where the water shell surrounding the protein is significantly reduced. Therefore, when the protein is sufficiently hydrated, interaction of negatively charged residues with surface silanolates-K^+^ ion pairs should be favored because of water mediation, while with fewer water molecules other interactions might prevail. Interestingly, the g(r)’s calculated for Model 3 (Figure 3d) show that the positively charged pepsin residues get actually quite close to the silanolates, suggesting a possible change of adsorption regime upon further dehydration of the system. In this respect, it is important to point out that on average 6 out of 8 silanolates are coordinated to K^+^: therefore, some negatively charged sites on the pore surface might be available for the binding of positively charged side chains of pepsin. Such an hypothesis is supported by the behavior of the hybrid system along the Model 3 trajectory: the rotational mobility observed for pepsin allowed for the transient approach of the positively charged Ile1 residues to the silanolates and suggested competition among positive pepsin side chains and potassium cations for adsorption on the deprotonated surface oxygens.

Analysis of the energy contributions of each component of the system, averaged over the 20 ns simulations and collected in Table 1, confirms the g(r)’s results. First of all, it should be noticed that the protein–silica channel pair interactions are very weak in all models. Significantly, protein–water attractive interactions become weaker in passing from the neutral to the charged silica mesopore because water molecules interact more favorably with the latter one, characterized by a higher hydrophilicity degree. In addition, the silica channel-K^+^ interactions become much stronger in the charged systems, while the silica channel–water pair interactions are nearly unaffected by the surface charge. The energies in the last line of Table 1 provide an estimate of the interaction between the mesopore and the droplet, (Pep + water + K^+^). The reported values indicate that the system with the charged surface is characterized by the strongest interaction, whereas the effect is much less significant in the neutral mesopore. As expected, and in keeping with the results obtained for the flat model surfaces, the dominant contribution to the nanodroplet–mesopore attraction arises from the interaction of the negatively charged silica matrix with potassium cations.

It is important to stress that the active site of the enzyme is not altered by the pore environment: its structure, characterized by small standard deviations, is close to those calculated for pepsin in water [63,64], on flat silica slabs [85], and also to crystallographic data [62], as demonstrated e.g., by the distance between the two Asp CG atoms of the catalytic dyad (Table 2). 

Also the average distances between the tip of the flexible loop (Gly76 CA) and the catalytic dyad (Asp215 CG), in the 1.0 ÷ 1.2 nm range, are in line with the crystal structure value (1.040 nm) [62]. The calculated standard deviation of ~0.2 nm is due to the flap mobility, which has also been observed for both pepsin in water solution [63,64] and pepsin in contact with flat silica surfaces [85], and is known to be fundamental for the catalytic activity of the enzyme because it favors the positioning of the substrate in the active site [31]. In addition, the average active site distances from the mesopore calculated for the three models are between 2.77 and 3.44 nm (Table 2) and indicate accessibility of substrate and water molecules to the active site of the enzyme. 

**Figure 4:**
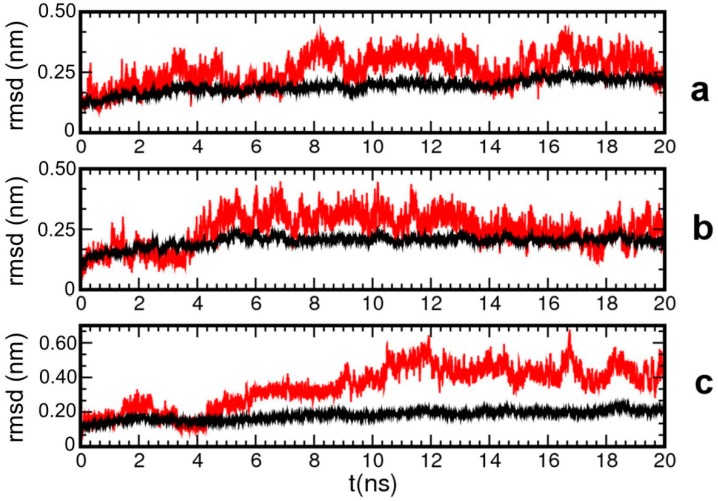
Time variation of root mean square distances (rmsd) with respect to the initial configuration calculated for all the pepsin residues (black line) and for the residues of the flap region (red line). Panel (**a**) simulation with the neutral channel (Model 1). Panel (**b**) simulation with the charged silica channel (Model 2). Panel (**c**) simulation with the charged channel and a lower number of water molecules (Model 3).

**Figure 5:**
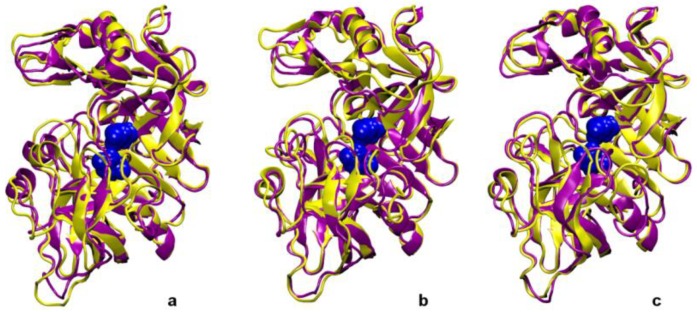
Superposition of the structure of pepsin at 0 ns (yellow) and at 20 ns (purple). Panel (**a**) simulation with the neutral channel. Panel (**b**) simulation with the charged channel. Panel (**c**) simulation with the charged channel and a lower number of water molecules.

Literature results from simulations on other enzymes interacting with inorganic matrices suggest that secondary and tertiary structures may be influenced by the support [16,36,45]. In order to detect possible modifications of the secondary and tertiary structure of pepsin as a consequence of its interaction with the pore, the root mean square distances (rmsd) of all pepsin residues with respect to the initial configuration were calculated (Figure 4, black lines). The average rmsd value over the 20 ns trajectory period is 0.192 ± 0.028 nm, 0.198 ± 0.023 nm, 0.177 ± 0.026 nm for Model 1, Model 2, and Model 3 respectively, and the largest contribution is due to the residues in the flap region (red lines), indicating therefore conservation of the enzyme structural features. Indeed, the size and shape of the protein do not change significantly upon confining the pepsin-containing droplet in a mesopore, even at partial dehydration conditions, as can be also deduced from the data in Table 2, and from the superposition of the 0–20 ns configurations (Figure 5). On the whole, and in analogy with what observed for flat silica surfaces, these results suggest therefore that the pepsin catalytic properties [62,86] are maintained in a water nanodroplet confined in mesoporous silicas. 

Investigations focused on proteins adsorption in mesoporous silicas have shown that the encapsulation process may be affected by different arrangements of the enzyme with respect to the inorganic support. Proteins may adopt a preferred orientation when binding to charged surfaces, for instance, the cytochrome c oxydase has been optimally loaded into cyano functionalized mesoporous silicas at pH 7, exposing its charged lysine groups towards the negatively charged regions of the inorganic matrix surface [87].

**Figure 6:**
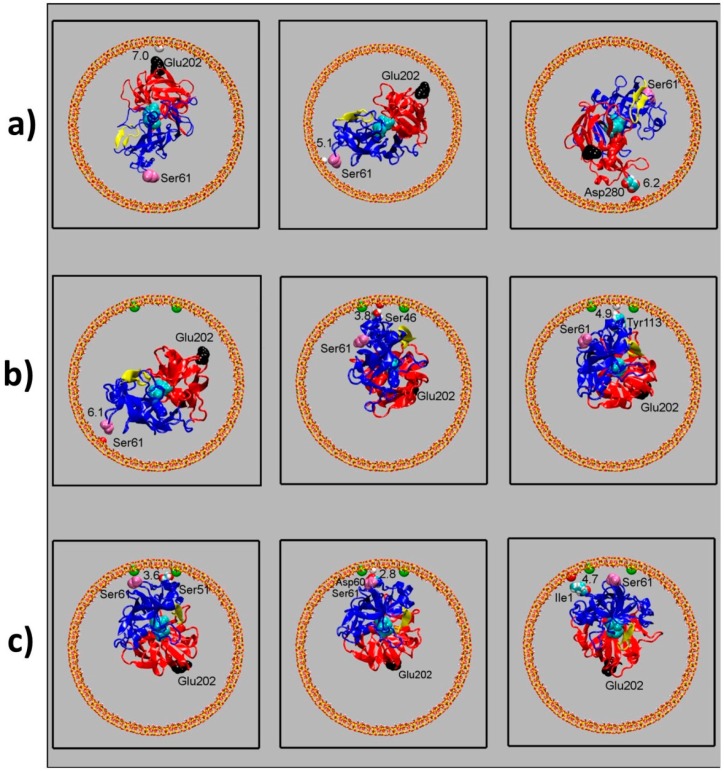
Snapshots representing different orientations of pepsin with respect to the surface. (**a**): simulation with the neutral channel (Model 1). (**b**): simulation with a negatively charged channel (Model 2). (**c**): simulation with a negatively charged silica channel and a smaller nanodroplet (Model 3). The mesopore (xy plane) is shown in stick representation; color: yellow for Si atoms, red for oxygen atoms and white for hydrogen atoms. The two pepsin domains are shown in red and blue respectively. The catalytic site is represented as cyan spheres, the flap region as a yellow ribbon. Ser61 (pink spheres) and Glu202 (black spheres) are the pepsin residues separated by the largest distance. The distance from the surface of the closest protein residue is reported for each configuration (in Å).

To detect whether pepsin adopts a preferential orientation inside the mesopore and investigate possible effects of the pore confinement on pepsin motion, the protein positioning and orientation with respect to the pore surface have been monitored along the simulations. Representative configurations sampled from the three trajectories are shown in Figure 6. The biodroplet does not adhere to the surface of a neutral mesopore (Model 1, Figure 6a): the protein undergoes both rotation and translation along the pore axis. In a negatively charged mesopore (Model 2, Figure 6b), pepsin can rotate because of the weak solution-mediated surface–protein interaction and does not show a preferential orientation with respect to the surface. In a smaller droplet such as the Model 3 (Figure 6c), this motion becomes more hindered but it still enables the enzyme to reorient. 

The observation that the calculated pair interactions between pepsin and the silica channel are very weak in all models suggests that the effects of pore flexibility on protein dynamics, and in particular on the catalytic dyad, should arguably be small. This supports a-posteriori our approximation of adopting a frozen atom model for the silica matrix, which allowed us to sample the confined nanodroplet behavior for a timeframe much longer compared to a fully flexible model. 

Nevertheless, the model presented herein could be surely improved. A first step might be the use of recently implemented Optimal Point Charge water models [88,89], that provide more accurate results in the properties of liquid water with respect to standard point-charge models. Thanks to their ability of reproducing the surface tension of water, OPC models have recently been employed in biomolecular simulations of lipid monolayers at the air−water interface [90]. A deeper improvement, however implying a significantly higher computational cost, could be achieved by releasing the frozen-atom-approximation of the silica channel, and, eventually, by using a flexible water molecule model.

## 3. Models and Methods

Molecular dynamics studies were performed on model systems composed by a water nanosized droplet containing the globular protein pepsin, inside a cylindrical silica mesopore, and included potassium cations in order to maintain electroneutrality of the system. Three distinct models were built, characterized by either different total electrostatic charge or hydration degree. Starting from a neutral hydroxylated silica pore model (Model 1), the inorganic support has been first deprotonated leading to a surface charge density compatible with pH 3.6 (Model 2) [31]; then, partial dehydration has been simulated by modelling a smaller protein-containing droplet in contact with a negatively charged mesopore. 

### 3.1. Building and Equilibration of the Mesopore

A nanometer-thick amorphous silica cylinder was chosen as a model for the pores of the SBA-15 material on the basis of experimental evidences indicating that these systems have arrays of pores of circular section in an amorphous silica matrix [31]. 

**Figure 7:**
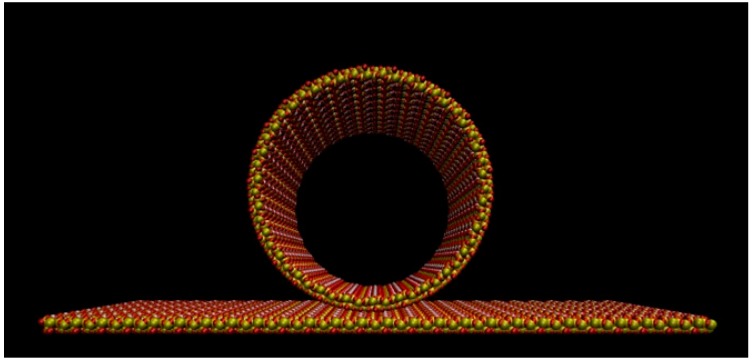
The rolling of the silica slab to form a cylindrical mesopore. The hydroxylated side of the slab becomes the inner surface of the pore. Atom colors: Si = yellow; O = red, H = white.

The silica ring shown in Figure 7 was prepared by “enrolling” a 2.008 nm × 26.104 nm sheet of amorphous silica and joining its ends. The length of the silica slab was chosen so as to obtain a ring of diameter compatible with that of the SBA-15 pores [31]. Such a nanometer-thick silica sheet, built on the basis of first-principles atomic models of hydroxylated silica surfaces [91,92,93,94,95], was the same adopted for modelling the flat surfaces described in Ref. [85].

The transformation from a slab to a cylinder has been performed with the following equations:

X′ = x cos(α) − y sin(α)(1)

Y′ = x sin(α) + y cos(α)(2)

**Scheme 1:**
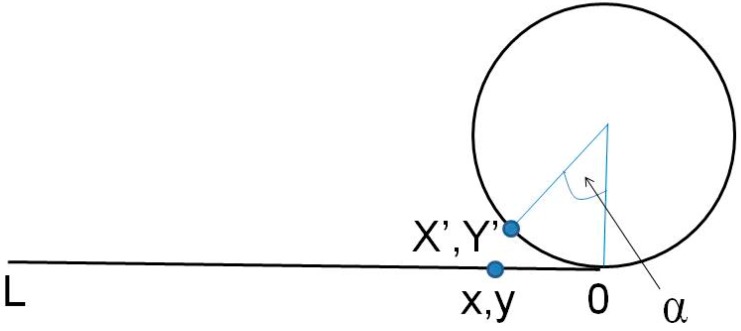
Geometrical scheme representing the α angle in the transformation equations relative to the transformation of the (x,y) coordinates on a slab of length L to the (X′,Y′) values on a cylinder of diameter L/π.

Z′ = z(3)

where (X′,Y′,Z′) represent the coordinates of an atom in the cylinder and (x,y,z) the coordinates of the same atom in the slab. The meaning of the angle α, whose value ranges from 0° to 360°, is sketched in Scheme 1.

**Figure 8:**
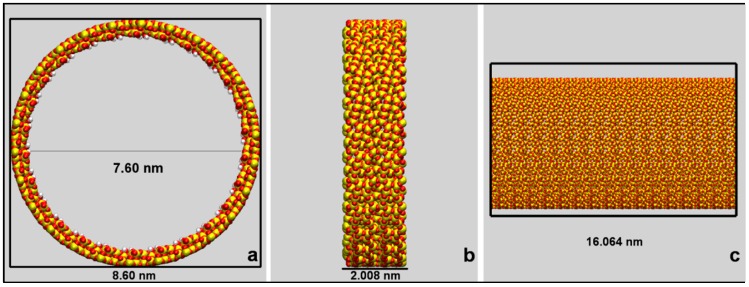
Graphical representations of the adopted cylindrical mesopore model. Atoms are represented as spheres with color codes: Si: yellow; O: red; H: white. Panel (**a**): circular section of the silica mesopore with an inner diameter of 7.60 nm and an external diameter of 8.60 nm. Panel (**b**): projection on the yz plane of the silica ring of 2.008 nm length. Panel (**c**) Projection on the yz plane of the cylindrical pore of 16.064 nm length. Atom colors: Si = yellow; O = red, H = white.

The guess configuration of the mesopore was built by replicating eight times along the z-axis the silica ring above described, which was characterized by an external diameter of 8.60 nm and a ~2 nm length (see Figure 8). 

**Figure 9:**
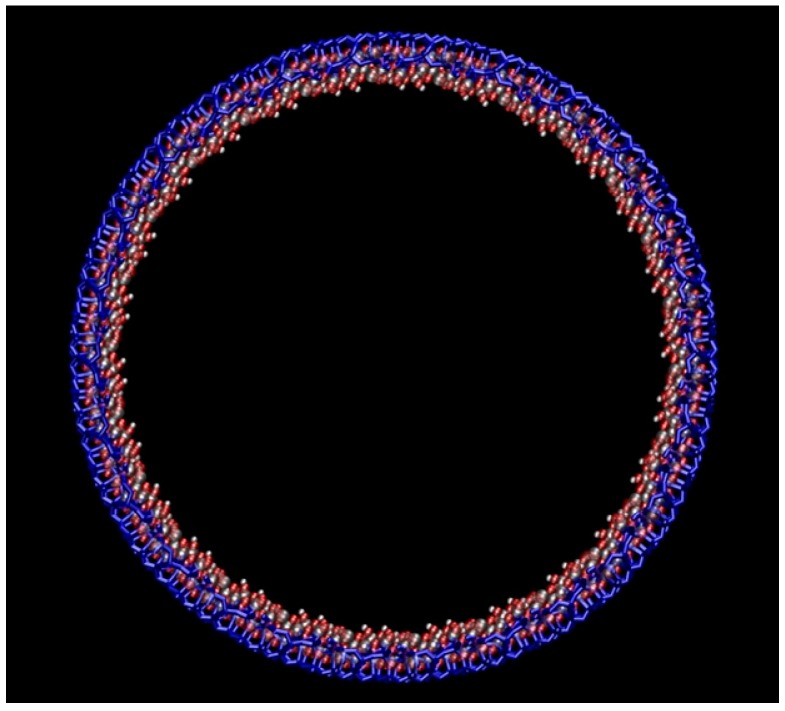
Optimization of the silica mesopore model. The blue lines represent the just-rolled silica cylinder. The optimized silica cylinder structure is represented by colored spheres: Si atom, gray spheres, O atoms, red spheres, H atoms, white spheres. The two structures are shown as projected along the z axis.

The silica cylinder has been successively optimized and the difference between the unrelaxed cylinder and the optimized structure is shown in Figure 9.

Defects at the ring junction, e.g., under- and over- coordinated Si and O atoms, as well as bond distances and angles distortions arising from forcing a curved geometry on the flat silica slab were eliminated by applying the following simulated annealing procedure [96]. The first step consisted in a molecular dynamics equilibration run of the neutral mesopore at 300 K, followed by quenching up to 20 K. Periodic boundary conditions were applied by adopting a supercell of 10.04 × 10.04 × 16.06 nm^3^. This scheme allows one to simulate a mesopore infinitely long in the *z* direction. Such an equilibration/annealing process, lasting 2 ns, has been repeated for a total of 20 ns. A final optimization was performed by adopting a quasi-quadratic minimization approach. This part of the work was carried out using the classical MD module of the CP2K suite of programs (see https://www.cp2k.org).

A mesoporous silica channel with a formal stoichiometry of [Si_6656_O_13312_∙(H_2_O)_832_] and 8.60 × 8.60 × 16.06 nm^3^ size was thus obtained. The mesopore was characterized by an internal circular section of 7.60 nm diameter and by a hydroxylated inner surface with silanol concentration of 3.97 OH/nm^2^. 

The internal force field adopted in the optimization and equilibration of the mesopore models is summarized in Table 3. Atoms were described by means of Lennard Jones pair potentials derived from the fitting of Buckingham type potentials [17].

**Figure 10:**
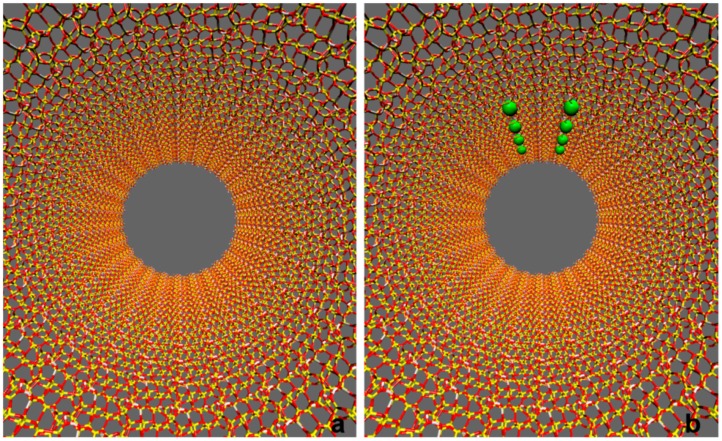
Graphical representations of the adopted silica mesopore model. Atom color codes: Si: yellow; O: red; H: white. Panel (**a**) Model 1: hydroxylated neutral surface. Panel (**b**) Model 2: surface with −8|e| charge. The green spheres on the charged surface mark the positions of the deprotonated silanol oxygens.

Two silica channel models were investigated, one electrically neutral (Model 1, Figure 10a) and one with surface charge of -8|e| (Model 2, Figure 10b). The surface charge density for Model 2 was −0.02|e|nm^−2^, a value compatible with the reported data for SBA-15 mesoporous silica matrices at pH 3.6 [31]. Since no information on the surface charge distribution of SBA-15 mesoporous systems was available, in the case of the charged mesopore the deprotonated silanols were arbitrarily positioned on a rectangular grid of 16 nm^2^ area on the inner pore surface. Specifically, the silanolates were arbitrarily arranged in a 2 × 4 square grid with SiO^−…^SiO^−^ separation of about 2 nm (Figure 10b). Such a choice is however corroborated by a modelling studies on the inner surfaces of a porous silica matrix, highlighting the formation of surface silanol rich patches [17,97], in line with the experimental evidence of hydrophobic and hydrophilic regions on the interior walls of mesoporous silicas [98]. 

### 3.2. Model Building and Simulation of the Nanobiodroplet Inside the Mesopore

Pepsin guess structure was based on the crystallographic structure of porcine pepsin (4PEP) [62], as described in Refs. [63,64,85]. The total electrostatic charge of pepsin was −6|e| and was defined by the protonation state of the protein side chains at pH = 3.6 [38], i.e., a pH which corresponds to the protein maximal loading in SBA-15 [31].

**Figure 11:**
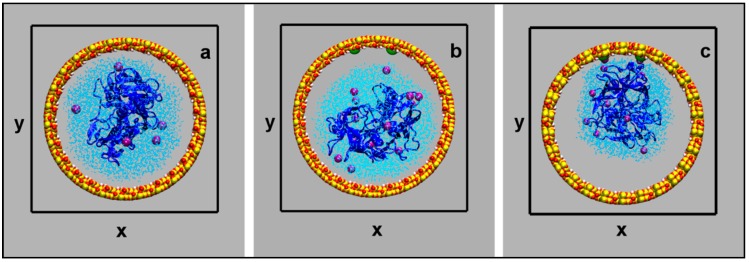
Projection of the simulation cells on the xy plane: neutral surface (**a**); charged surface (**b**,**c**). The partially dehydrated model is shown in picture c. The water droplet (light blue lines) contains pepsin (blue ribbon structure) and potassium cations (purple spheres). The silica slab atoms are represented as spheres (color codes: Si, yellow; O red; H, white; silanolates, green. The snapshot represents the starting configuration for Model 1 (**a**), Model 2 (**b**), and Model 3 (**c**). The orthorhombic unit cell of 10.04 × 10.04 × 16.06 nm^3^ is represented as black lines.

The simulation system consisted of a total of 37,709 atoms for Model 1 (including 3506 water molecules) and 37,685 atoms for Model 2 (including 3498 water molecules). Such a choice of the number of water molecules enables one to build a droplet of 6.50 × 6.80 × 7.30 nm^3^ size, which is compatible with the diameter of the mesopore models, but still large enough to solvate the protein. The center of mass of pepsin was located in the middle of the silica channel, and at about 4 nm from the negatively charged region of the pore inner surface in Model 2. A partially dehydrated model (Model 3) was built starting from the final configuration of the Model 2 simulation and removing a 1 nm thick water shell from the protein-containing droplet. The simulation cell for Model 3 consisted of 34,574 atoms (including 2461 water molecules). Periodic boundary conditions were applied in three dimensions by adopting an orthorhombic unit cell of 10.04 × 10.04 × 16.06 nm^3^ (see Figure 11). 

Pepsin was described by the Charmm 22 Force Field [99], the rigid TIP3P model was used for water [100], and long-range interactions were treated using a particle-mesh Ewald summation scheme [101]. The silica channel atoms were kept fixed and interacted with the water molecules and the protein via van der Waals forces and long-range electrostatic forces. The charges of the mesopore atoms were +2.4|e| for Si, −1.2|e| for O, and +0.6|e| for H. The charge of the deprotonated surface oxygens was −1.6|e|. Electroneutrality of the full system was obtained by adding K^+^ cations (6K^+^ for Model 1, and 14 K^+^ for Model 2). The K^+^ concentration for Model 1 and 2 was ~4 × 10^−2^ M and ~1 × 10^−1^ M respectively, similar to the values reported for the preparation of the fully hydrated SBA-15-pepsin composite [31].

The choice of the adopted Force Field (FF) for water deserves some comments. Indeed, a number of recent and more sophisticated water FFs, including flexible and polarizable water models, have appeared in the literature, [102] which provide accurate results for liquid water [103,104] and for water confined in aluminosilicate microporous systems [103]. A steep barrier to the application of these improved models to biomolecular simulations, which typically involve a substantial number of water molecules, is their extra computational complexity [102]. Recently, Optimized Point Charge models have been developed [88,89] and applied, e.g., in lipid monolayer simulations [90]. This class of approaches includes a 3-point water model which is more accurate than conventional water models at a still convenient computational cost [88]. Here, however, we adopted the TIP3P FF because it is largely used in simulations of biosystems and is incorporated in many available molecular dynamics packages. Also importantly, such a choice allows us for a straightforward comparison with previous studies involving pepsin [63,64,85]. 

A further approximation of our models derives from fixing the silica matrix atoms. However, such an approximation affects mainly the fast vibrational motion of the silanols’ OH. On the other hand, also the fast vibrational motion of the water molecules is missing in the rigid water models commonly used in biosystems. This has as a practical consequence that the short time (0 to 1 ps) behavior of our system is less accurate than its long time behavior (ns scale), which is however the regime we are interested in. 

The simulations were performed in the NVT ensemble at 300 K with an integration time step of 1 fs. Equilibration was checked by monitoring protein dimensions, distances between selected residues, and distances between pepsin residues and slab atoms; small standard deviations were obtained in all cases. Equilibration runs of 15 ns were performed in all cases, while presented results were averaged over production trajectories of 20 ns. The adopted simulation time is much longer than the ones typically adopted for water nanodroplet simulations [66,84], in particular no signal of water molecule evaporation from the droplet was detected. 

For the three models, the NAMD suite of programs was used [105]. Graphical representation of the models were created with the VMD code [106].

## 4. Conclusions

By modelling a water nanodroplet containing the negatively charged protein pepsin confined either into a neutral or a negatively charged silica mesopore, with diameter comparable to SBA-15 pores, relevant aspects of its behavior were uncovered. 

We found that the nanobiodroplet adheres only to negatively charged patches of the pore surface, without any direct contact among protein and surface atoms because the interaction is mediated by water molecules and by the counter ions bound to the negative surface sites. Moreover, in this non-physiological environment, pepsin does not undergo conformational changes and may easily reorient by rotation. Last but not least, its active site is fully accessible to substrates and maintains the spatial arrangement needed for catalytic activity. 

Overall, our findings imply that confinement of the pepsin-containing nanodroplet in a mesopore does not significantly affect structure and motion of protein. Also, in the case of the neutral mesopore, the detected flow of the protein-containing nanodroplet along the channel clearly shows that mere confinement effects could not lead to stable pepsin-mesoporous silica composites. Recently, nanobiodroplets, i.e., protein-containing water nanodroplets have been generated in a volatile electrospray environment. Our modeling study indicates that nanobiodroplets might be stabilized and eventually stored for experimental investigation inside silica mesopores. Moreover, the hydrophobic (neutral) channel allows for the droplet flow in the directionally controlled environment provided by the mesoporous silica structure. 

## Figures and Tables

**Table 1:** Pair interaction energies (in kJ mol^−1^) for Model 1, Model 2, and Model 3, respectively. Standard deviations in parenthesis.

Pair	Model 1	Model 2	Model 3
Pep–water	−24,230 (565)	−23,882 (611)	−23,326 (624)
Pep–silica channel	−54 (4)	268 (92)	469 (59)
Pep–K^+^	−1665 (351)	−2548 (377)	−3234 (389)
Pep–K^+^ per ion	−276 (59)	−184 (25)	−230 (29)
Water–silica channel	−80 (21)	−255 (293)	−151 (226)
Water–K^+^	−2946 (343)	−6418 (552)	−5724 (477)
Water–K^+^ per ion ^a^	−490 (59)	−460 (38)	−410 (33)
Silica channel–K^+^	−121 (54)	−2536 (46)	−3247 (247)
Pep–(silica channel + water + K^+^)	−25,949 (469)	−26,376 (444)	−25,882 (510)
Silica channel–(pep + water + K^+^)	−80 (21)	−2561 (96)	−2632 (84)

^a^ This entry should be intended as K^+^ hydration energy.

**Table 2:** Calculated average inter-atomic distances (nm) between pepsin residues with standard deviations. Distances from the 4PEP crystal structure (nm) are reported for comparison.

System	Asp215-Asp32	Gly76-Asp215	Asp215-Surface	Ser61-Glu202
Model 1	0.486 ± 0.045	0.880 ± 0.155	3.444 ± 0.134	6.388 ± 0.104
Model 2	0.520 ± 0.077	1.060 ± 0.206	2.773 ± 0127	6.430 ± 0.096
Model 3	0.522 ± 0.089	1.187 ± 0.165	2.927 ± 0096	6.300 ± 0.140
Pep–crystal structure	0.510	1.040	-	59

**Table 3:** Lennard-Jones pair potential parameters used for the optimization of the mesopore model.

Atom-Atom Pair	ε (kJ/mol)	Rmin (nm)
Si–Si	0	0
Si–O	109.04	0.167
O–O	1.979	0.328
O–H	0.946	0.145
Si–H	0.0874	0.135
H–H	0.0249	0.101
